# Implementation of an Integrated, Clinical Decision Support Tool at the Point of Antihypertensive Medication Refill Request to Improve Hypertension Management: Controlled Pre-Post Study

**DOI:** 10.2196/70752

**Published:** 2025-04-11

**Authors:** John Charles Matulis 3rd, Jason Greenwood, Michele Eberle, Benjamin Anderson, David Blair, Rajeev Chaudhry

**Affiliations:** 1 Division of Community Internal Medicine, Geriatrics and Palliative Care, Mayo Clinic, 200 1st St. SW, Rochester, MN, 55905, United States, 1 5072845278; 2 Department of Family Medicine, Mayo Clinic Health System, Eau Claire, WI, United States; 3 Department of Pharmacy, Mayo Clinic, Rochester, MN, United States

**Keywords:** clinical decision support systems, population health, hypertension, electronic health records

## Abstract

**Background:**

Improving processes regarding the management of electronic health record (EHR) requests for chronic antihypertensive medication renewals may represent an opportunity to enhance blood pressure (BP) management at the individual and population level.

**Objective:**

This study aimed to evaluate the effectiveness of the eRx HTN Chart Check, an integrated clinical decision support tool available at the point of antihypertensive medication refill request, in facilitating enhanced provider management of chronic hypertension.

**Methods:**

The study was conducted at two Mayo Clinic sites—Northwest Wisconsin Family Medicine and Rochester Community Internal Medicine practices—with control groups in comparable Mayo Clinic practices. The intervention integrated structured clinical data, including recent BP readings, laboratory results, and visit dates, into the electronic prescription renewal interface to facilitate prescriber decision-making regarding hypertension management. A difference-in-differences (DID) design compared pre- and postintervention hypertension control rates between the intervention and control groups. Data were collected from the Epic EHR system and analyzed using linear regression models.

**Results:**

The baseline BP control rates were slightly higher in intervention clinics. Postimplementation, no significant improvement in population-level hypertension control was observed (DID estimate: 0.07%, 95% CI −4.0% to 4.1%; *P*=.97). Of the 19,968 refill requests processed, 46% met all monitoring criteria. However, clinician approval rates remained high (90%), indicating minimal impact on prescribing behavior.

**Conclusions:**

Despite successful implementation, the tool did not significantly improve hypertension control, possibly due to competing quality initiatives and high in-basket volumes. Future iterations should focus on enhanced integration with other decision support tools and strategies to improve clinician engagement and patient outcomes. Further research is needed to optimize chronic disease management through EHR-integrated decision support systems.

## Introduction

The contemporary electronic health record (EHR) enables patients and pharmacies to request chronic medication renewals, which comprise significant in-basket volumes that primary care teams must address. Typical EHR renewal request interfaces provide clinicians with limited information when addressing the refill request [1]. For patients not seen recently, it takes time for conscientious clinicians to review relevant electronic data to ensure that the requested renewal is appropriate for that patient’s care plan. Ideally, physicians or a staff member would always review relevant data, including the date of the last clinician visit, presence of up-to-date laboratory monitoring, and prior documentation, to confirm that the medication is appropriately addressing important outcomes such as blood pressure, blood glucose, or cholesterol control [2]. However, in the context of rapidly increasing in-basket volumes, such diligence is unlikely in the absence of a supportive system, as primary care providers (PCP) struggle to “get through one’s in-basket” [3].

The EHRs have long been recognized as powerful tools that can provide real-time decision support to assist clinicians in cognitively demanding and tedious tasks. Examples of clinical decision support integrated into EHR medication prescription platforms include alerts when prescribing medications to which a patient may be allergic, medications that may have an unsafe interaction with another prescribed medication, and medications that would generate significant out-of-pocket costs for the patient [4-6]. Hypertension, the most common chronic condition treated in primary care [7], has shown that EHR-integrated applications, as described by Funes et al [8], can improve clinician workflows in chronic disease management. In addition, the American Heart Association and the American Medical Association have called upon primary care teams to better leverage protocols and team-based care to improve hypertension control [9]. This study is particularly timely, as the management of hypertension when patients are not in the office is becoming increasingly relevant for quality reporting and reimbursement [10]. As more health care systems transition to value-based payment [11], the management of uncontrolled hypertension will become a higher priority in clinical practice. Given this context, prescription renewal requests provide a valuable opportunity for the primary care team to ensure safe prescribing practices and improved management of this difficult-to-treat condition.

In this study, we describe the implementation and evaluation of an EHR-integrated clinical decision support tool, eRx HTN Chart Check, which incorporates relevant structured data necessary for monitoring hypertension management into the prescription renewal request interface. We aimed for this tool to improve care provided to both individual patients with hypertension and population-level control of hypertension.

## Methods

### Study Setting: Mayo Clinic Health System, Northwest Wisconsin and Mayo Clinic, Rochester, MN

The division of Family Medicine, Mayo Clinic Health System (MCHS)-Northwest Wisconsin (NWWI) employs 108 providers, including 80 physicians and 28 advanced practice providers (APPs), caring for approximately 96,000 patients in 11 primary care clinics across a five-county area centered around Eau Claire, Wisconsin. The Mayo Clinic division of Community Internal Medicine (CIM) in Rochester Minnesota, employs 75 PCPs (60 physicians, 15 APPS) caring for 35,000 patients at 5 primary care sites within a 40-mile radius of the community of Rochester, MN. Both sites are part of Mayo Clinic, a not-for-profit health care system providing both “destination” care for serious and complex issues and primary care for local communities.

The control groups consisted of the Department of Family Medicine in Southeast Minnesota, which employs 57 physicians and 88 APPs who care for 115,000 patients, and the Department of Internal Medicine in MCHS-Southwest Wisconsin, which employs 7 physicians and 3 APPs who care for 7,000 patients. All primary care practices included in this study have designated the management of uncontrolled hypertension as a quality management priority during the intervention period.

### eRx HTN Chart Check Project and Team

Before the eRx HTN chart check project, automated prescription renewal requests were sent directly to PCPs through the EHR in-basket without additional clinical information. Specifically, refill requests did not include relevant clinical information such as the patient’s most recent blood pressure, most recent office visit, or relevant laboratory results.

In January 2022, a multidisciplinary team representing Mayo Clinic campuses in Rochester, MN, and Mayo Clinic Health System-NWWI was assembled. The project team aimed to integrate clinical decision support into the electronic prescription renewal process to improve clinical efficiency, provider experience, and the quality of care provided. The project team included a project manager, 3 physician leaders from the two participating sites, nursing leaders, a pharmacist informaticist, and a practice administrator.

### Hypertension Chart Check Protocol Development

Internal EHR user data showed that PCPs spent significant in-basket time reviewing and approving chronic prescription medications. Antihypertensive medication renewals were selected as the initial target for integrated clinical decision support, given the importance of hypertension management in population health and the need for an interface to facilitate an efficient review of relevant clinical information. This included hypertension control, the date of the most recent office visit or blood pressure reading on file, and appropriate laboratory monitoring for specific medications.

The eRx HTN Chart Check integrated key clinical data (BP readings, visit dates, and laboratory results) directly into the prescription renewal interface. A pass/fail grading system flagged values based on predefined thresholds: BP readings from the past 24 months and laboratory results from the past 12 months. A detailed view, available to the clinician via hyperlink, displayed up to three recent values, with standing laboratory orders and future laboratory orders. For patients without an active hypertension diagnosis, the system excluded recent BP readings, and for those of childbearing age, it ensured no positive pregnancy test within the past 12 months.

The team, with selective input from Mayo Clinic experts and practice resources, determined appropriate monitoring and developed protocols for commonly prescribed classes of antihypertensive medications (Multimedia Appendix 1). The pass/fail criteria were determined through an iterative process involving a multidisciplinary team of physician leaders, pharmacists, nursing informaticists, and EHR specialists. Criteria were based on established clinical guidelines for hypertension management, including recommended monitoring intervals for BP measurements and laboratory assessments for antihypertensive medication safety. Adjustments were made based on Mayo Clinic expertise coupled with historical prescribing patterns to balance clinical rigor with practical usability. While no formal external validation was conducted, the criteria were aligned with national guidelines and vetted through internal stakeholder feedback to ensure clinical appropriateness.

**Figure 1.**
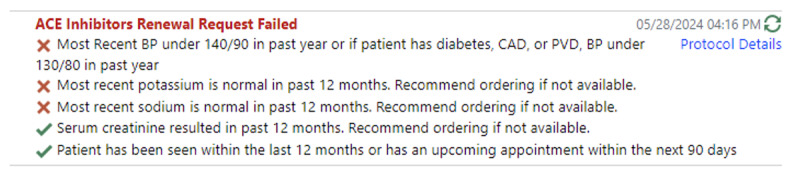
Pass/fail display in renewal requests. This figure illustrates how the eRx HTN Chart Check tool presents structured clinical data within the prescription renewal interface. Green checkmarks (✔) indicate that the required clinical data (e.g., recent BP reading, appropriate laboratory monitoring) meet predefined thresholds; red marks (❌) flag missing or outdated data, prompting clinician review before approving renewal.

After completion of medication selection and protocol development, relevant discrete data for the medication renewal protocol were converted into a visual pass/fail display, which would appear directly below the renewal request message (Figure 1).

Preimplementation efforts (April 2022) included clinician presentations and educational materials. The tool was launched at MCHS-NWWI on July 15, 2022, and at Rochester CIM on August 1, 2022. No modifications were made postimplementation, but periodic clinician meetings reinforced tool usage.

The eRX HTN Chart Check tool was adopted across both practice environments with minimal levels to workflows. Some physicians raised concerns about whether the eRx HTN Chart Check failure implied an expectation that the physician was responsible for addressing outstanding items. Clarification was provided to clinician teams that this was not the intent; rather, the information was presented only for the prescribing physician’s awareness. In our subjective experience, most clinicians impacted by the change found the additional information available to be helpful in efficiently completing these renewal requests, although this finding was not quantitatively evaluated.

### Study Design and Sample

We evaluated our eRx HTN Chart Check intervention using a DID design. The outcomes in the intervention group were collected through monthly hypertension control metrics, spanning 6.5 months before and 5.5 months after the intervention’s implementation period, which occurred between June and August 2022. The specific dates of the intervention timelines are outlined in Table 1. The DID design allows estimation of the intervention effect by comparing the change in outcomes over time between the intervention group relative to the control group, accounting for secular trends. Visual inspection of these trends indicated similar trajectories, supporting the validity of the parallel trend’s assumption. Additionally, no significant interaction between time and group was observed in the preintervention period when tested using regression analysis, further reinforcing this assumption. No patients or prescribers at either intervention or control sites were excluded.

**Table 1.** Timelines for baseline data collection, intervention implementation, and postimplementation data collection. The intervention was implemented sequentially at different sites, with clinician education provided before each launch. Postimplementation surveillance included monitoring clinician engagement and assessing hypertension control trends.

Study phase	Timelines for data collection	Intervention group	Control group	Activities
Preintervention	January-June 2022	NWWI^a^ FM+^b^RST^c^ CIM^d^	MCHS^e^ CIM+RST FM	Intervention design, iterative feedback
Implementation	July 15, 2022	NWWI FM	FM RST	Education, real-time support
Implementation	August 1, 2022	RST CIM	SWWI CIM	Education, real-time support
Post Implementation	August 2022-December 2022	NWWI FM + RST CIM	MCHS CIM+ RST FM	Surveillance

a NWWI: Northwest Wisconsin.

b FM: Family Medicine.

c RST: Rochester.

d CIM: Community Internal Medicine.

e MCHS: Mayo Clinic Health System.

### Ethical Considerations

The Mayo Clinic Institutional Review Board deemed this project as a quality improvement initiative and exempted it from formal review. All data were maintained on encrypted, password-protected systems behind the organization’s firewall, with access limited to authorized project team members only. No patient identifiers were retained in the final analysis or manuscript, and all reporting is based on deidentified, aggregate information to ensure confidentiality and compliance with institutional privacy standards.

### Primary Outcomes

The primary outcome was the average percentage of patients with a recorded BP reading of <140/90 before and after the study period. This BP target of <140/90 was selected in accordance with the Minnesota Community Measurement program and National Quality Foundation standards [12,13]. The 6-month follow-up period for outcome assessment was chosen to allow the prescriber to evaluate the possible impact of subsequent BP management after an antihypertensive prescription refill request.

### Secondary Outcomes

Secondary outcomes reflect measures related to antihypertensive refill volumes and the rates of clinician approval for medications prepared via the eRx HTN Chart Check protocol.

### Data Sources and Analysis

The unit of analysis for the primary outcome was all eligible patients within each practice who had a formal diagnosis of hypertension in the EHR, compatible with definitions of the Minnesota Community Measurement program’s definitions [12]. For secondary outcomes, the unit of analysis included patients whose eRx HTN Chart Check renewals were completed during the study period. We ascertained patient-level data on hypertension control through an internal EHR data platform used for quality improvement. We extracted eligible prescription renewal encounter data from the Epic EHR Cogito resources [14] (Workbench, SlicerDicer, and direct data queries with either internal or external visualization tools) to determine clinician response rates to eligible chart check encounters.

### Statistical Methods

To assess changes in BP control across practices, we calculated the proportion of all eligible patients with a recorded BP at treatment goal (<140/90) within the preceding 12 months, consistent with National Quality Foundation guidelines [13]. We calculated these proportions by both study period and group and conducted a DID analysis using aggregated clinic-level proportions as the dependent variable and exposure to the intervention as the independent variable. We calculated the between-group differences using a linear regression model to adjust for the clustering of data within clinics. The model included fixed effects for time and group to control for baseline differences and time trends. The intervention effect was reported as the mean difference in proportions, with 95% CIs and *P* values. Statistical significance was defined as *P*<.05, and analyses were performed using Blue Sky Statistics (version 10.3.4; BlueSky Statistics LLC). Secondary outcomes were analyzed using descriptive statistics.

## Results

### Baseline Characteristics of Intervention Patients

We identified 15,591 patients in the NWWI division of Family Medicine and 11,766 patients in the Rochester CIM cohort in our baseline dataset. Relevant study characteristics are included in Table 2.

**Table 2.** Baseline preintervention patient characteristics.

Clinical site	Patients with essential hypertension (n)	Average number of total medications	Eligible patients taking antihypertension medication (N=15,591), n (%)	Average number of antihypertension medications (n)	Average age (years)	White patients, n (%)	English-speaking patients, n (%)	Smoker patients, n (%)
NWWI-FM^a^	15,591	11.6	14,439 (93)	2.1	66	15,159 (97)	15,441 (99)	1766 (11.3)
RST-CIM^b^	11,766	13.1	10,709 (91)	1.7	69	15,314 (88)	16,393 (94)	1096 (9.3)
SWWI-CIM^c^	3368	14.1	3154 (94)	2.1	70.9	3267 (97)	3354 (99)	242 (7.2)
RST-FM^d^	24,782	11.8	22,166 (89)	1.8	61.2	22,303 (90)	24,073 (97)	1437 (5.8)

a NWWI: Northwest Wisconsin-Family Medicine.

b RST-CIM: Community Internal Medicine.

c SWWI: Southwest Wisconsin-Family Medicine.

d RST-FM: Family Medicine.

We then performed a DID analysis to evaluate the impact of eRx HTN Chart Check implementation on clinic-level compliance proportions. The baseline compliance proportion was higher in the intervention clinics compared to the control clinics (difference: 3.32%, 95% CI 0.5%-6.2%; *P*=.02). Across all clinics, there was no significant change in compliance proportions from the baseline to the intervention period (difference: 1.29%, 95% CI: −1.6% to 4.2%; *P*=.37). The DID estimate, reflecting the interaction between intervention group and time period, was not statistically significant (difference: 0.07%, 95% CI −4.0% to 4.1%; *P*=.97). These findings suggest that the intervention did not result in a significant additional improvement in compliance proportions compared to the control group.

**Figure 2.**
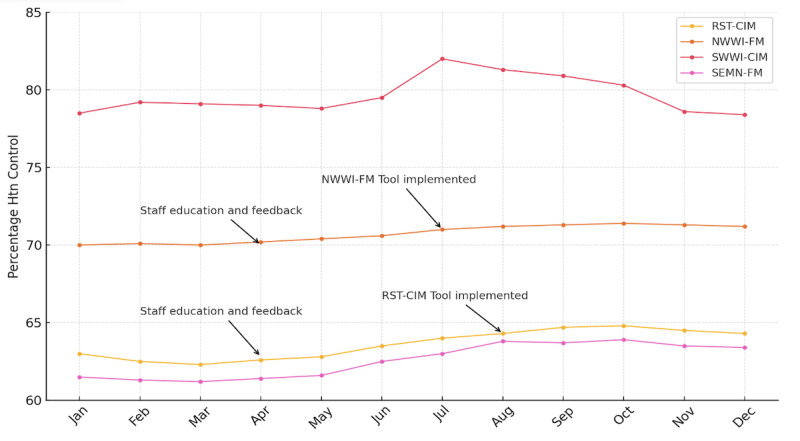
Hypertension control by site. Htn: hypertension; RST-CIM: Rochester-Community Internal Medicine; NWWI-FM: Northwest Wisconsin-Family Medicine; SWWI: Southwest Wisconsin-Community Internal Medicine; SEMN-FM: Southeast Minnesota-Family Medicine.

Figure 2 presents a line chart depicting BP changes before, during, and after the intervention across all analyzed patient cohorts. No significant DID was observed in compliance proportion changes between groups over the study period.

### Secondary Outcomes: Chart Check Completion Rates and Subsequent Blood Pressure Re-Measurement

Secondary outcomes are displayed in Table 3. A total of 19,968 refills for antihypertensive medications were completed through the eRx HTN Chart Check process during the study period. Among these, 9,256 (46%) prescriptions “passed” or had all current monitoring information (Figure 2) available at the time of renewal.

**Table 3.** Secondary outcomes.

Clinical site	Refills submitted via chart check (n)	Number of refills “passed protocol”, n (%)	Refills approved by clinician, n (%)
NWWI-FM^a^	10,771	5018 (47)	9225 (86)
RST-CIM^b^	9197	4238 (46)	8748 (95)
Combined intervention group	19,968	9256 (46.4)	17,973 (90)

a NWWI: Northwest Wisconsin-Family Medicine.

b RST-CIM: Rochester-Community Internal Medicine.

## Discussion

### Principal Findings

In this evaluation of the implementation of the eRx HTN Chart Check tool, we found that its use was not associated with significant improvements in population-level hypertension control when compared with practices in the same health care system that were unexposed to the intervention. While the eRx HTN chart check tool identified that approximately 50% of patients requesting prescription renewals had incomplete hypertension management parameters, most prescriptions were still filled, suggesting that this tool may not have significantly changed prescriber behavior.

There are several possible explanations that may account for why the eRx HTN Chart Check intervention did not result in improved population-level hypertension control compared with unexposed practices, as hypothesized. First, the management of hypertension was a concurrent quality priority across all health care system sites at the time of this intervention, and several ongoing efforts for patient outreach and enhanced office care and blood pressure rechecks. These efforts were already underway, including efforts to better capture home BP readings in the EHR and proactive follow-up for patients with hypertension, among others. Given the heightened awareness of hypertension management, clinicians may have relied on other interventions to address hypertension management rather than the eRx HTN Chart Check tool, which may have been used primarily to ensure appropriate medication monitoring. Additionally, changes in clinical guidelines, medication costs, insurer coverage, increasing telehealth utilization, and health care consumerism could have played a role in shaping hypertension outcomes independent of our intervention. While the DID analysis adjusted for baseline differences and time trends, the presence of multiple simultaneous initiatives limited our ability to isolate the specific impact of the eRx HTN Chart Check intervention. Second, in the post-COVID period, primary care in-basket volumes increased substantially across our practice and other clinics nationwide [15]. This increased workload may have resulted in less clinician attention being allocated to prescription renewal requests. This premise is supported by the remarkably prescription approval rates reported in our secondary outcomes, suggesting that time constraints may have led clinicians to prioritize efficiency over detailed clinical review when addressing these requests. Third, the eRx HTN Chart Check intervention only appeared for refill requests that originated from the pharmacy, meaning it may not have effectively captured or been applied to all prescription renewal requests, including those sent directly from patients, thereby limiting the overall reach of this intervention.

This study applies a novel EHR clinical decision support (CDS) tool aimed at promoting chronic disease management through extant processes of routine medication renewals. The successful implementation of the eRx HTN Chart Check tool demonstrates that routine care processes such as chronic disease medication renewals can present opportunities for the integration of clinical decision support and current clinical workflows, as well as standardizing the information gathered across sites in a group practice. The eRx HTN Chart Check tool can be enhanced, adapted, and combined with other interventions to address important population health priorities such as control of hypertension. Additionally, other important outcomes, such as prescribing safety, appropriate monitoring, care escalation, patient engagement, provider satisfaction, and cognitive load should be explored in subsequent assessments of integrated CDS in the contemporary primary care setting.

This study is subject to the typical limitations of this study design, including the possibility of selection bias due to lack of randomization, regression to the mean, the Hawthorne effect, and time-related biases such as misattribution. These limitations were significantly mitigated by the inclusion of similar control groups practicing in comparable environments and with similar quality priorities. However, it is important to note that the findings may have limited generalizability due to the geographic, health care system, and patient population characteristics of the included sites. In health care systems with fewer resources, different clinician workflows, or alternative staffing models, the adaptation of this tool may vary. Additionally, patient populations served by community health centers or safety-net institutions may have different levels of medication adherence and follow-up needs. Therefore, future research should evaluate how this tool functions in other nonintegrated health care settings. Moreover, while our primary outcome focused on population-level hypertension control, we did not examine patient-level variables that could influence blood pressure outcomes, such as medication adherence, follow-up visit rates, or patient-reported measures of disease burden. These factors are critical in understanding hypertension management but were not captured in our dataset, which was limited to structured EHR-derived clinical data. Future research should incorporate patient-centered metrics—including follow-up care, medication adherence, and qualitative assessments of patient and provider experiences—to provide a more comprehensive evaluation of CDS interventions in chronic disease management.

Future efforts should be closely tied with other team-based efforts to maximize efficiency and leverage important patient touchpoints, such as prescription renewals, to improve care delivery and standardize practices enabling cross-clinic site support. Machine learning and artificial learning may serve as valuable future enhancements in potentially aiding clinicians in targeted interventions [16]. Additional efforts are needed to understand how organizations can optimally support PCPs in using valuable tools such as the eRx HTN Chart Check. Additionally, further alignment between add-on tools such as the eRx HTN Chart Check and other CDS tools such as Practice Advisories, Health Maintenance Navigators, and outreach platforms should be encouraged. This alignment would ensure a uniform approach, easier maintenance, and facilitate seamless updates as guidelines and protocols change.

### Conclusion

In this controlled pre-post study evaluating the implementation of a CDS tool for antihypertensive medication refill requests, we found that implementation of this tool into existing workflows did not improve population-level BP control compared with groups unexposed to the intervention. Despite the lack of significant improvements in hypertension control, this study highlights the potential for EHR-based decision support tools to impact chronic disease management. Aligning the eRx HTN Chart Check with other EHR-based interventions such as automated patient outreach, remote BP monitoring integration, and machine learning may improve its utility and adoption. Future research should explore multimodal decision support strategies, clinician education initiatives, and the impact of interoperability with broader population health tools to optimize hypertension management in diverse clinical settings. Future research should focus on optimizing these interventions, exploring alternative implementation strategies, and expanding their application to other chronic conditions.

## Supplementary material

10.2196/70752 Multimedia Appendix 1 Hypertension chart check protocol.

## Abbreviations

APP: advanced practice provider
BP: blood pressure
CDS: clinical decision support
CIM: Community Internal Medicine
DID: difference-in-differences
EHR: electronic health record
MCHS: Mayo Clinic Health System
NWWI: Northwest Wisconsin
PCP: primary care provider
