# Supplementary material for: Implementation of an Integrated, Clinical Decision Support Tool at the Point of Antihypertensive Medication Refill Request to Improve Hypertension Management: Controlled Pre-Post Study
Source: JMIR Med Inform. 2025 Apr 11;13:e70752. doi: 10.2196/70752 (PMC12007840; doi:10.2196/70752)
Supplement: Multimedia Appendix 1 [file medinform-v13-e70752-s001.docx]

Key

Display Text: Text that ordering clinician will view when refill request enters in-basket

Summary Report Information: source data informing display text

Filter Criteria: additional filter applied to source data

**ACE-Inhibitors:**

| **Display Text** | **Summary Report Information** | **Filter Criteria** |
| --- | --- | --- |
| Serum creatinine resulted in past 12 months. Recommend ordering if not available. | [Last serum creatinine](epic:EPIC?REPORT&RecordSummary&ETX%5e13537%5e%5e1) in 24 months |  |
| Most recent potassium is normal in past 12 months. Recommend ordering if not available. | [Last serum potassium](epic:EPIC?REPORT&RecordSummary&ETX%5e13538%5e%5e1) in 24 months |  |
| Most recent sodium is normal in past 12 months. Recommend ordering if not available. | [Last serum sodium](epic:EPIC?REPORT&RecordSummary&ETX%5e13538%5e%5e1) in 24 months |  |
| eGFR decrease greater than 10% between most recent results in the last 2 years. Recommend provider review. | Last 3 eGFR in 24 months | eGFR has decreased > 10% for the most recent value in the last two years |
| Most Recent BP under 140/90 in past year or if patient has diabetes, CAD, or PVD, BP under 130/80 in past year | [Last](epic:EPIC?REPORT&RecordSummary&ETX%5e13539%5e%5e1) 3 ambulatory visit BP |  |
| No active pregnancy on record |  | Female of childbearing age* |
| No positive pregnancy test in past 12 months |  | Female of childbearing age* |
| Patient has been seen within the last 12 months or has an upcoming appointment within the next 90 days | [Recent](epic:EPIC?REPORT&RecordSummary&ETX%5e16857%5e%5e1) encounters (12 months), future encounters (90 days), and unscheduled appointments |  |
| Medication is Active on patient medication list |  | [Will](epic:EPIC?REPORT&RecordSummary&LGL%5e3352%5e%5e1) display only if medication is not currently on the Patient’s medication list |

**Beta-Blockers:**

| **Display Text** | **Summary Report Information** | **Filter Criteria** |
| --- | --- | --- |
| Patient has been seen within the last 12 months or has an upcoming appointment within the next 90 days | [Recent](epic:EPIC?REPORT&RecordSummary&ETX%5e16857%5e%5e1) encounters (12 months), future encounters (90 days), and unscheduled appointments |  |
| No active pregnancy on record |  | Female of childbearing age* |
| No positive pregnancy test in past 12 months |  | Female of childbearing age* |
| Most Recent BP under 140/90 in past year or if patient has diabetes, CAD, or PVD, BP under 130/80 in past year | [Last](epic:EPIC?REPORT&RecordSummary&ETX%5e13539%5e%5e1) 3 ambulatory visit BP |  |
| Serum creatinine resulted in past 12 months. Recommend ordering if not available. | [Last serum creatinine](epic:EPIC?REPORT&RecordSummary&ETX%5e13537%5e%5e1) in 24 months | Atenolol order |
| Medication is Active on patient medication list |  | [Will](epic:EPIC?REPORT&RecordSummary&LGL%5e3352%5e%5e1) display only if medication is not currently on the Patient’s medication list |

**Angiotensinogen Receptor Blockers**

| **Display Text** | **Summary Report Information** | **Filter Criteria** |
| --- | --- | --- |
| Patient has been seen within the last 12 months or has an upcoming appointment within the next 90 days | [Recent](epic:EPIC?REPORT&RecordSummary&ETX%5e16857%5e%5e1) encounters (12 months), future encounters (90 days), and unscheduled appointments |  |
| No active pregnancy on record |  | Female of childbearing age* |
| No positive pregnancy test in past 12 months |  | Female of childbearing age* |
| Most Recent BP under 140/90 in past year or if patient has diabetes, CAD, or PVD, BP under 130/80 in past year | [Last](epic:EPIC?REPORT&RecordSummary&ETX%5e13539%5e%5e1) 3 ambulatory visit BP |  |
| Serum creatinine resulted in past 12 months. Recommend ordering if not available. | [Last serum creatinine](epic:EPIC?REPORT&RecordSummary&ETX%5e13537%5e%5e1) in 24 months |  |
| Most recent potassium is normal in past 12 months. Recommend ordering if not available. | [Last serum potassium](epic:EPIC?REPORT&RecordSummary&ETX%5e13538%5e%5e1) in 24 months |  |
| Most recent sodium is normal in past 12 months. Recommend ordering if not available. | [Last serum sodium](epic:EPIC?REPORT&RecordSummary&ETX%5e13538%5e%5e1) in 24 months |  |
| eGFR decrease greater than 10% between most recent results in the last 2 years. Recommend provider review. | Last 3 eGFR in 24 months | eGFR has decreased > 10% for the most recent value in the last two years |
| Medication is Active on patient medication list |  | [Will](epic:EPIC?REPORT&RecordSummary&LGL%5e3352%5e%5e1) display only if medication is not currently on the Patient’s medication list |

**Calcium Channel Blocker:**

| **Display Text** | **Summary Report Information** | **Filter Criteria** |
| --- | --- | --- |
| Patient has been seen within the last 12 months or has an upcoming appointment within the next 90 days | [Recent](epic:EPIC?REPORT&RecordSummary&ETX%5e16857%5e%5e1) encounters (12 months), future encounters (90 days), and unscheduled appointments |  |
| No active pregnancy on record |  | Female of childbearing age* |
| No positive pregnancy test in past 12 months |  | Female of childbearing age* |
| Most Recent BP under 140/90 in past year or if patient has diabetes, CAD, or PVD, BP under 130/80 in past year | [Last](epic:EPIC?REPORT&RecordSummary&ETX%5e13539%5e%5e1) 3 ambulatory visit BP |  |
| Active on medication list |  | [Medication not active on med list](epic:EPIC?REPORT&RecordSummary&LGL%5e3352%5e%5e1) |

**Diuretics:**

| **Display Text** | **Summary Report Information** | **Filter Criteria** |
| --- | --- | --- |
| Serum creatinine resulted in past 12 months. Recommend ordering if not available. | [Last serum creatinine](epic:EPIC?REPORT&RecordSummary&ETX%5e13537%5e%5e1) in 24 months |  |
| Most recent potassium is normal in past 12 months. Recommend ordering if not available. | [Last serum potassium](epic:EPIC?REPORT&RecordSummary&ETX%5e13538%5e%5e1) in 24 months |  |
| Most recent sodium is normal in past 12 months. Recommend ordering if not available. | [Last serum sodium](epic:EPIC?REPORT&RecordSummary&ETX%5e13538%5e%5e1) in 24 months |  |
| eGFR decrease greater than 10% between most recent results in the last 2 years. Recommend provider review. | Last 3 eGFR in 24 months | eGFR has decreased > 10% for the most recent value in the last two years |
| Most Recent BP under 140/90 in past year or if patient has diabetes, CAD, or PVD, BP under 130/80 in past year | [Last](epic:EPIC?REPORT&RecordSummary&ETX%5e13539%5e%5e1) 3 ambulatory visit BP |  |
| No active pregnancy on record |  | Female of childbearing age* |
| No positive pregnancy test in past 12 months |  | Female of childbearing age* |
| Patient has been seen within the last 12 months or has an upcoming appointment within the next 90 days | [Recent](epic:EPIC?REPORT&RecordSummary&ETX%5e16857%5e%5e1) encounters (12 months), future encounters (90 days), and unscheduled appointments |  |
| Medication is Active on patient medication list |  | [Will](epic:EPIC?REPORT&RecordSummary&LGL%5e3352%5e%5e1) display only if medication is not currently on the Patient’s medication list |

**Vasodilators:**

| **Display Text** | **Summary Report Information** | **Filter Criteria** |
| --- | --- | --- |
| Patient has been seen within the last 12 months or has an upcoming appointment within the next 90 days | [Recent](epic:EPIC?REPORT&RecordSummary&ETX%5e16857%5e%5e1) encounters (12 months), future encounters (90 days), and unscheduled appointments |  |
| Most Recent BP under 140/90 in past year or if patient has diabetes, CAD, or PVD, BP under 130/80 in past year | [Last](epic:EPIC?REPORT&RecordSummary&ETX%5e13539%5e%5e1) 3 ambulatory visit BP |  |
| Medication is Active on patient medication list |  | [Will](epic:EPIC?REPORT&RecordSummary&LGL%5e3352%5e%5e1) display only if medication is not currently on the Patient’s medication list |

*Childbearing age is 10-55 years
